# Sporotrichosis in Older Adults: A Cohort Study of 911 Patients from a Hyperendemic Area of Zoonotic Transmission in Rio de Janeiro, Brazil

**DOI:** 10.3390/jof9080804

**Published:** 2023-07-30

**Authors:** Rachel da Silva Ribeiro Gomes, Antonio Carlos Francesconi do Valle, Dayvison Francis Saraiva Freitas, Priscila Marques de Macedo, Raquel de Vasconcellos Carvalhaes Oliveira, Rodrigo Almeida-Paes, Rosely Maria Zancopé-Oliveira, Maria Clara Gutierrez-Galhardo

**Affiliations:** 1 Laboratory of Clinical Research in Infectious Dermatology, Evandro Chagas National Institute of Infectious Diseases, Oswaldo Cruz Foundation, Av. Brasil, 4365, Manguinhos, Rio de Janeiro 21040-900, Brazil; 2 Laboratory of Epidemiology, Evandro Chagas National Institute of Infectious Diseases, Oswaldo Cruz Foundation, Rio de Janeiro 21040-900, Brazil; 3 Laboratory of Mycology, Evandro Chagas National Institute of Infectious Diseases, Oswaldo Cruz Foundation, Rio de Janeiro 21040-900, Brazil

**Keywords:** sporotrichosis, older adults, zoonotic, *Sporothrix brasiliensis*, osteoarticular form, itraconazole, terbinafine, cryosurgery

## Abstract

Generally, older people tend to suffer from more severe infections than younger adults. In addition, there are accumulations of comorbidities and immune senescence in some cases. This cohort study evaluated the clinical and epidemiological characteristics of older adults (≥60 years old) with sporotrichosis. The cohort consisted of 911 patients with a median age of 67 years, most of whom were female (72.6%), white (62.1%), and afflicted with comorbidities (64.5%). The lymphocutaneous form occurred in 62% of the patients, followed by the fixed form (25.7%), cutaneous disseminated form (8.9%), and extracutaneous/disseminated forms (3.3%). In this study, we draw attention to the frequency of osteoarticular involvement (2.1%) secondary to skin lesions such as osteomyelitis and/or tenosynovitis. A clinical cure was achieved in 87.3% of cases. Itraconazole was used in 81.1% of cases, while terbinafine was used in 22.7% of cases, usually in low doses. Survival analysis showed that the median treatment time was 119 days, and the multiple Cox model demonstrated that the presentation of a black coloration and diabetes was associated with a longer treatment time required to establish a cure. Therefore, these subgroups should be monitored more closely to reduce possible difficulties during treatment. It would be interesting to conduct more studies analyzing older adults with sporotrichosis from different geographic areas to better comprehend the disease in this group.

## 1. Introduction

Sporotrichosis is the most common subcutaneous mycosis across the globe, and it is caused by dimorphic fungi belonging to the genus *Sporothrix*. The traditional route of infection is traumatic inoculation via plant thorns and decaying wood. Zoonotic transmission, especially from infected cats, and fungal conidial inhalation from the environment are other routes of infection [1,2].

Cat-transmitted sporotrichosis due to *Sporothrix brasiliensis* is a major public health problem in Brazil, with Rio de Janeiro serving as its epicenter and a spreading profile across the country over the last 25 years [2,3,4,5]. Outside of Brazil, confirmed outbreaks of cat-transmitted *S. brasiliensis* have been described in Argentina, Paraguay, Chile, and the United Kingdom [5,6,7]. This is an emerging species and the most virulent of the *Sporothrix* genus, as observed in an experimental animal model [8]. Severe clinical forms (mainly infecting immunosuppressed individuals) leading to hospitalizations and deaths are associated with this agent [9,10,11].

In general, older adults are more likely to contract more severe infections than younger adults due to their accumulation of comorbidities and immune senescence [12,13]. The latter is characterized by a decline in immune response (both innate and acquired) and a pro-inflammatory condition known as “Inflamm-aging” involving an increase in interleukin-6 and tumor necrosis factor [14,15]. Elderly frailty syndrome, which is more pronounced after 80 years of age, is a consequence of the decline of many physiological systems, resulting in vulnerability to changes in health status triggered by stressful events [16].

Older people constitute an important cohort of those affected by zoonotic sporotrichosis in Rio de Janeiro, where over 70% of transmission occurs at home [4]. The task of caring for animals is often assigned to this group, which is related to the fact that they spend more free time at home. The objective of this study was to evaluate the clinical and epidemiological characteristics, as well as the evolution and outcomes, of a cohort of older adults with sporotrichosis from a reference center in a region of zoonotic transmission in Rio de Janeiro.

## 2. Materials and Methods

### 2.1. Study Location, Design, and Patients

We conducted an observational and retrospective study on a cohort of patients aged 60 years or older who had been diagnosed with sporotrichosis from 1999 to 2020. This study was carried out at the Evandro Chagas National Institute of Infectious Diseases (INI/FIOCRUZ), which has been a primary reference centre for sporotrichosis cases in the hyperendemic area of Rio de Janeiro, Brazil, since 1998. The inclusion criteria were an age of 60 years and above and culture-proven sporotrichosis from any clinical specimen [2]. Patients without clinical data that would allow for a clinical assessment and an adequate therapeutic response were excluded. This study was approved by the Institutional Research Ethical Committee under the number 53337121.3.0000.5262.

### 2.2. Definitions

Sporotrichosis was classified in the following forms: lymphocutaneous, fixed cutaneous, cutaneous disseminated, unifocal extracutaneous (single site of involvement or two contiguous sites), and multifocal/disseminated (two or more non-contiguous sites) [11].

Regarding the unifocal extracutaneous and multifocal/disseminated forms, organ involvement was defined as the isolation of *Sporothrix* spp. from the respective clinical specimens, such as sputum or a mucosal swab, or by utilizing a clinical picture compatible with laboratory and imaging examinations for patients with fungus isolated from the skin.

A clinical cure was defined as the healing of cutaneous or mucosal lesions and the disappearance or stabilization of pulmonary (with negative findings from a mycological examination of sputum) or bone lytic lesions. Clinical relapse was defined as the reemergence of lesions after healing, for which new fungal specimens were isolated from a lesion or when there was a therapeutic response without laboratory confirmation. Clinical reinfection was defined as the appearance of new lesions associated with another exposure to *Sporothrix* sp. after the cure that is not anatomically related to the previous lesions.

### 2.3. Patient Management

The initial evaluation of all patients with sporotrichosis admitted to the INI follows a standardized protocol that includes clinical evaluation and routine laboratory tests (complete blood count and levels of glucose, creatinine, urea, alkaline phosphatase, gamma-glutamyl transpeptidase, and serum transaminases). Serology for sporotrichosis using the ELISA (enzyme-linked immunosorbent assay) technique [17] is performed whenever possible on the first blood sample collected after admission. Subsequent laboratory exams are carried out according to clinical evolution every 2–3 months, in the case of longer treatments, and to verify any adverse effect of the medication, in case of clinical suspicion. The first treatment choice is itraconazole, which is administered orally at a dosage of 100–200 mg/day, or, in the case of a contraindication, alternative drugs such as terbinafine 250–500 mg and potassium iodide solution [18,19]. They are used until a clinical cure is achieved. Higher doses of antifungals are used when there is no improvement or worsening of the clinical picture after at least 2 months of treatment. Amphotericin B deoxycholate or amphotericin lipid complex are used in standardized doses in osteoarticular, meningeal, and pulmonary forms, depending on their severity, and in cutaneous forms unresponsive to oral antifungal treatment [11]. Once the clinical condition is stabilized, amphotericin is suspended, and then oral antifungal drugs are prescribed to complete a minimum of 12 months of treatment in case of osteoarticular, meningeal, and pulmonary forms [18].

Cryosurgery is indicated for cutaneous lesions due to an incomplete response to oral therapy or the impossibility of using oral antifungal agents as previously described [20]. Electrosurgery is also performed when there is no response to cryosurgery. Re-evaluations are conducted monthly until a cure is achieved. Once a clinical cure has been achieved, the treatment is discontinued, and the patients are followed up for 1–3 months until they are discharged.

### 2.4. Data Collection

Clinical, epidemiological, and laboratory data were collected from patients’ medical records. These data were anonymized and re-identified to protect the patients’ privacy and confidentiality.

### 2.5. Statistical Analysis

The variables were stored in a database (Excel) and analyzed with the survival package of the open-access program R version 4.2.1. Considering that different age groups can lead to different clinical and evolutionary profiles, we had previously defined the following groups for analysis: group 1, namely, patients aged ≥60 and <80 years, and group 2, namely, those aged ≥80 years. Due to the great ethnic diversity in Brazil, we defined the skin color/race variable as corresponding to white or black (brown/mixed-black and black). Ethnicity/color was established by the administrative staff at the time of registration in the institute until the year 2005; subsequently, this information was self-reported by the patients according to their ancestry.

Frequencies, percentages, and summary measures such as median and interquartile range (IQR) were calculated. In the calculation of frequencies in terms of percentages, valid cases were considered, and missing data were excluded. To compare the categories of quantitative variables to the data regarding cured and non-cured cases, the T-test or Mann–Whitney test (depending on the distribution pattern) and Pearson’s χ2 test (chi-square) or Fisher exact test, for the qualitative variables, were used. All hypothesis tests were performed considering a significance level of 5%. In addition, whenever possible, 95% confidence intervals have been provided.

Exploratory survival analysis was used to investigate treatment time until reaching a favorable outcome (cure). The treatment time was calculated using the difference between the dates at the end and at the beginning of a treatment. Censoring was defined with reference to abandonment and death dates. The Kaplan–Meier (KM) method was used to estimate the survival curves stratified by qualitative variables. A multiple Cox model was built using the variables with variation between the KM survival curves in the Log-Rank test (*p* < 0.05) and the theoretical relevance (age). Due to the lack of proportionality of the risks in the Schoenfeld residuals, the stratification of the baseline risk according to the clinical form variable in the multiple model was considered. The model effects were interpreted as crude and adjusted Hazard Ratios (HRs) of the single and multiple covariate Cox models, respectively. HR > 1 indicates that more events resulted in a cure (or took less time to cure), HR < 1 indicates that fewer events resulted in a cure (or take a longer time to cure), and HR~0 indicates that there is no difference between the groups.

## 3. Results

**Figure 1:**
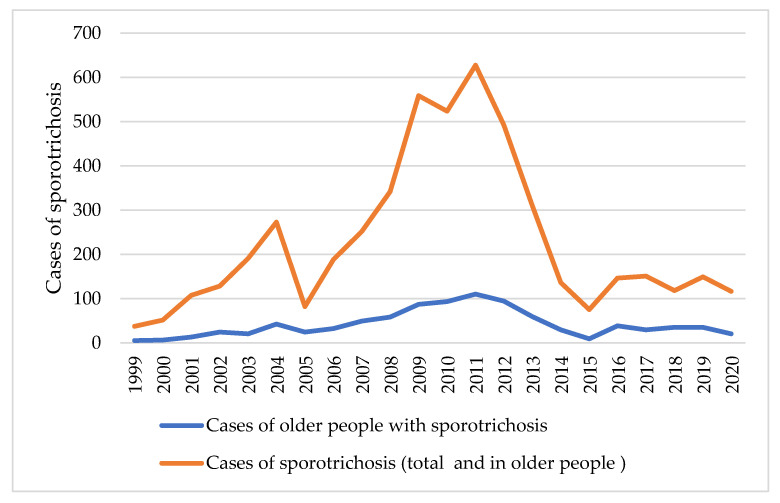
Annual cases of sporotrichosis (total and cases involving older people) at INI/FIOCRUZ from 1999 to 2020.

From 1999 to 2020, 5056 patients were diagnosed with sporotrichosis at INI/FIOCRUZ, of which 1002 patients were 60 years old and above. A total of 91 patients were excluded, resulting in a dataset of 911 patients (18% of all sporotrichosis patients). Among them, 834 (91.6%) patients were in group 1, and 77 (8.3%) patients were in group 2. The annual frequency of the total number of cases of sporotrichosis and the cases involving older people can be seen in Figure 1. The median number of cases of sporotrichosis per year was 150 patients, with 33.5 cases of older people with sporotrichosis.

### 3.1. Socio-Demographic and Epidemiological Characteristics

The distribution of characteristics is shown in Table 1. Most patients were female (*n* = 666, 73.1%), and there was a higher predominance of females (*n* = 66, 85.7%) in group 2 when compared with group 1 (*n* = 600, 71.9%) (*p*-value = 0.013). The age of the patients ranged from 60 to 95 years, with a median age of 67 (IQR = 63–73) years old. Eleven patients were over 87 years old. Of the 853 patients with available skin color/race data, 62.1% (*n* = 530) were white and 37.9% were black (*n* = 323), and there were no differences between groups 1 and 2.

Most of the 911 cases (*n* = 823, 90.3%) originated in high-density areas of zoonotic sporotrichosis in Rio de Janeiro and a group of cities in its metropolitan region. As for their occupations, most patients were out of the labor market (*n* = 602, 66.1%). Regarding possible sources of infection, 785 (88.2%) patients reported contact with cats. Scratching or biting was reported by 508 (57.1%) patients. Contact with plants was reported in 53 (6.0% of 890 patients) cases, 52 (5.8% of 890 patients) of which involved patients who could not account for the source of the infection.

### 3.2. Clinical Forms

**Figure 2:**
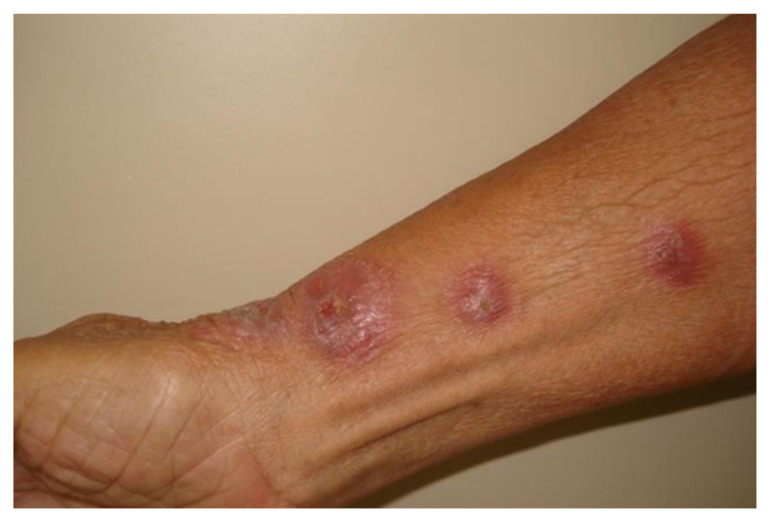
Lymphocutaneous sporotrichosis an 83-year-old woman. Typical erythematous nodule-ulcerated lesions following the lymphatic vessels on the left forearm.

Of 905 patients, 62% (*n*= 562) patients presented the lymphocutaneous form (Figure 2), 25.7% (*n* = 233) presented the fixed cutaneous form, 8.9% (*n* = 81) presented the cutaneous disseminated form, and 3.3% (*n* = 30) patients presented the extracutaneous/disseminated form (Table 1).

**Figure 3:**
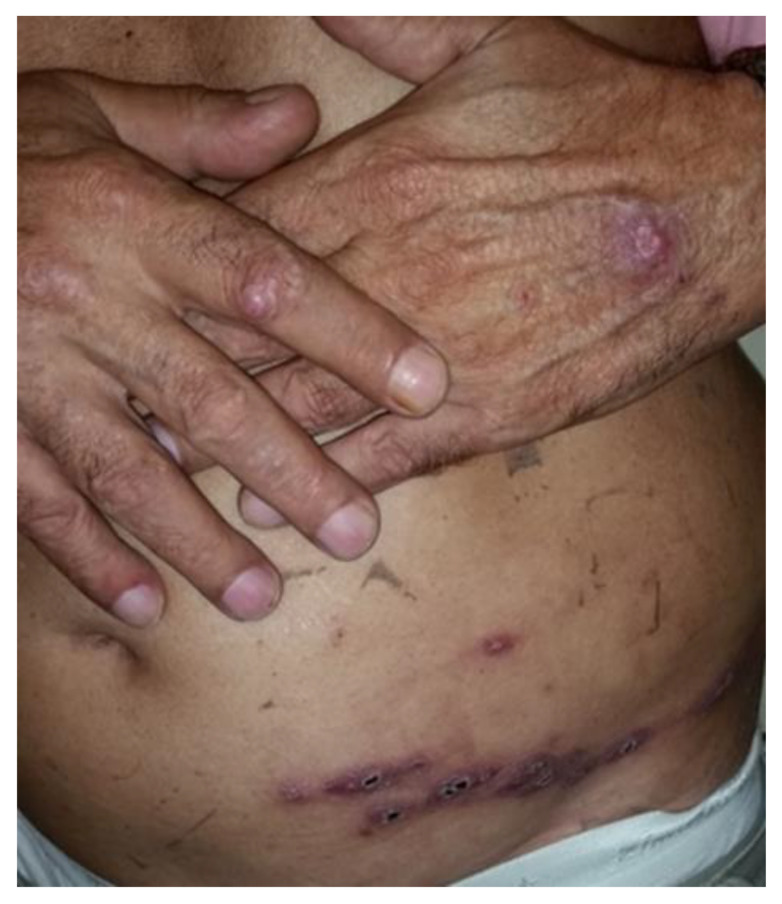
Cutaneous disseminated sporotrichosis in a 66-year-old man. This patient presents nodular-ulcerated lesions on the dorsa of their hands and a lymphocutaneous/zosteriform distribution on their abdomen.

The lesions were more frequent on the upper limbs (*n* = 726, 79.7%), followed by the lower limbs (*n* = 151, 16.6%), head (*n* = 61, 6.7%), and trunk (*n* = 45, 4.9%). Seventy-two patients (7.9%) had lesions on more than one body segment (Figure 3). There were no differences between the clinical forms and the studied groups.

**Figure 4:**
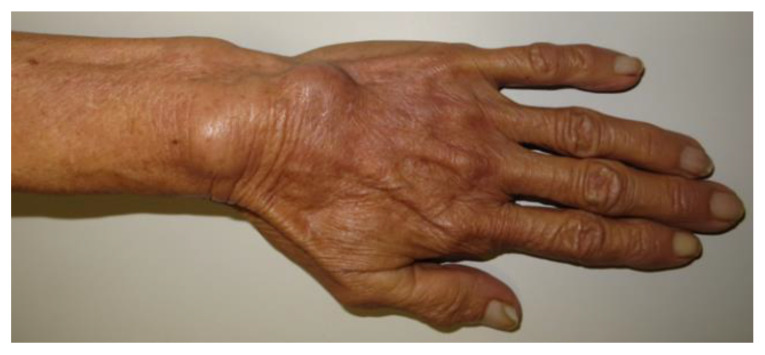
Lymphocutaneous sporotrichosis with tenosynovitis involvement in a 75-year-old woman. Two nodules/masses on the dorsum of the left wrist.

**Figure 5:**
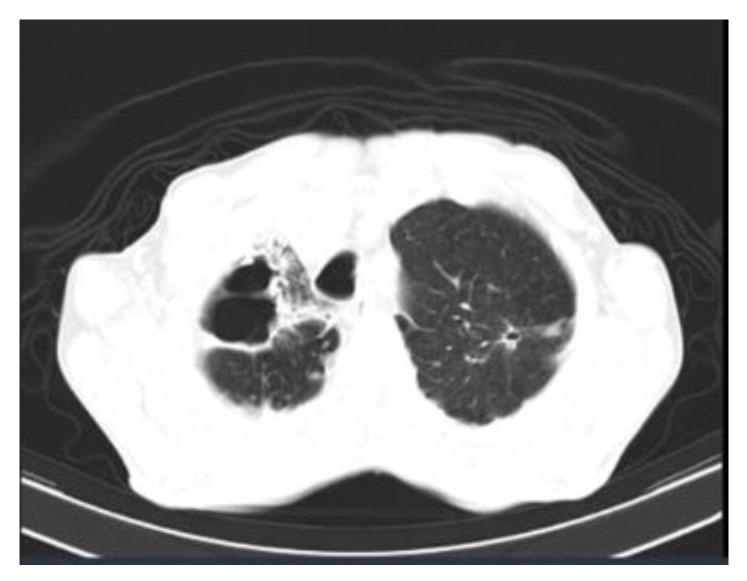
Pulmonary sporotrichosis in a 63-year-old man. Nonenhanced chest computed tomography showing, among other alterations, thick-walled cavities with irregular margins in the right lower lobe associated with architectural distortion, fibrotic opacities, and bronchiectasis. There are also linear opacities with a fibrotic appearance and bronchiectasis in the apicoposterior segment of the left upper lobe.

**Figure 6:**
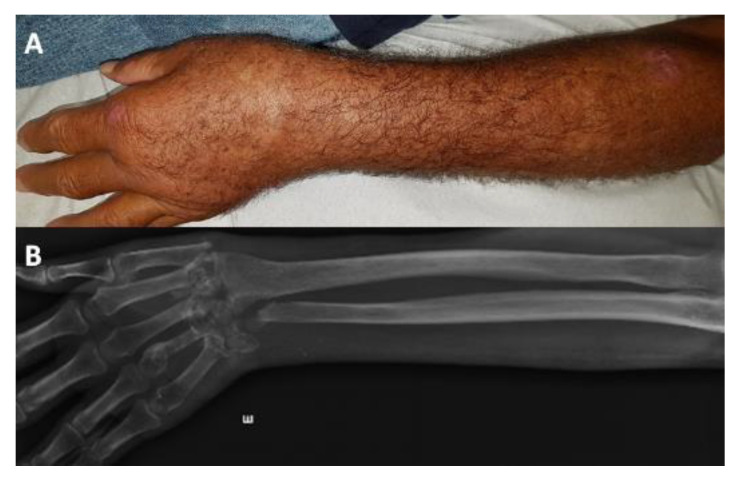
Disseminated sporotrichosis (cutaneous, tenosynovitis associated, and osteoarticular) in a 76-year-old man. (**A**) Scars on the skin of the left forearm along with swelling of the wrist. (**B**) Radiography showing extensive morphostructural alterations of the carpal bones, with loss of their individualization and wrist joint relationships in addition to deformity of the distal end of the 4th metacarpal and bone destruction of the distal epiphysis of the ulna.

Table 2 describes the clinical presentations of patients classified as possessing unifocal extracutaneous (Figure 4) and multifocal/disseminated extracutaneous lesions (Figure 5). All but two patients with osteomyelitis presented bone lesions on their hands (Figure 6).

### 3.3. Hypersensitivity Reactions

**Figure 7:**
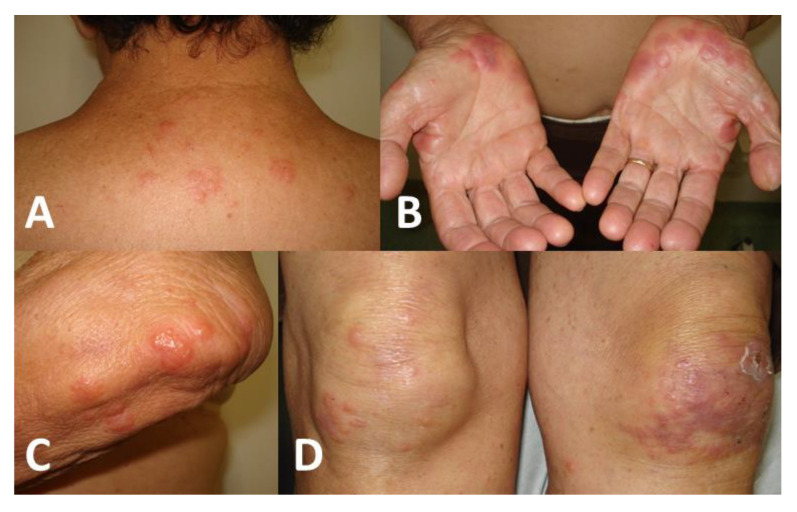
Fixed cutaneous sporotrichosis with hypersensitivity reaction (Sweet’s syndrome) in a 78-year-old woman. Hypersensitivity reaction: pruritic cutaneous papules and plaques on the dorsum of the trunk (**A**), palms of the hands (**B**), elbows (**C**), and knees (**D**). Sporotrichosis lesion: small exulcerated lesion on the left knee (**D**).

Hypersensitivity reactions associated with sporotrichosis occurred in 25 (2.8%) cases, the details of which are as follows: erythema multiforme (12, 48.0%), erythema nodosum (4, 16.0%), Sweet’s syndrome (2; 8.0%), and undefined (7, 28.0%). The fixed cutaneous form (*n* = 11) was the most frequent presentation associated with these hypersensitivity reactions (Figure 7), followed by the lymphocutaneous form (*n* = 8), disseminated cutaneous form (*n* = 3), disseminated form (lymphocutaneous and ocular), and unifocal extracutaneous form (ocular) (*n* = 1, respectively). For one patient, the clinical form was missing. When separated into groups, patients aged 80 and above presented a significantly lower frequency of hypersensitivity reactions (1/77, 1.3%) compared to group 1 (24/834, 2.9%) (*p* value = 0.00009).

### 3.4. Comorbidities and Concomitant Medication Use

Among the 873 patients who provided answers regarding whether they had comorbidities, 636 (76.0%) reported comorbidities. The number of comorbidities *per* patient is described in Table 1.

When we compared the presence of comorbidities between the groups, there was no difference between the groups. The median value of comorbidities *per* patient and IQR in both groups were similar (1, 1–2).

The most reported comorbidity was systemic arterial hypertension (*n* = 478, 75.2%), followed by diabetes mellitus (*n* = 163, 25.6%), dyslipidemia/metabolic alterations (*n* = 48, 7.5%), and cardiovascular diseases (*n* = 46, 7.2%) (Supplementary Table S1). Among the 636 patients with comorbidities, 601 (94.4%) used medication to treat them, with 169 (26.6%) using one type of medication, 148 (23.8%) patients taking two medications, 120 (13.9%) patients using three medications, and 164 (25.8%) patients on four to twelve medications. The median number of medications taken *per* patient in the groups and the IQR values were similar (2, 1–4).

Sixteen (1.7%) patients had immunosuppressive conditions or used immunosuppressive medications. Six patients had a history of alcohol abuse; six were using immunosuppressive agents (methotrexate, prednisone, or azathioprine) for rheumatoid arthritis and idiopathic thrombocytopenic purpura; and four were people living with HIV (PLHIV) who were on antiretroviral therapy. Four patients (4) who abused alcohol presented disseminated (2), disseminated cutaneous (1), or pulmonary (1) sporotrichosis. The other patients presented the lymphocutaneous (8) or fixed cutaneous form (4), all but one of which were in group 1.

### 3.5. Blood Alterations in Routine Laboratory Examinations

**Figure 8:**
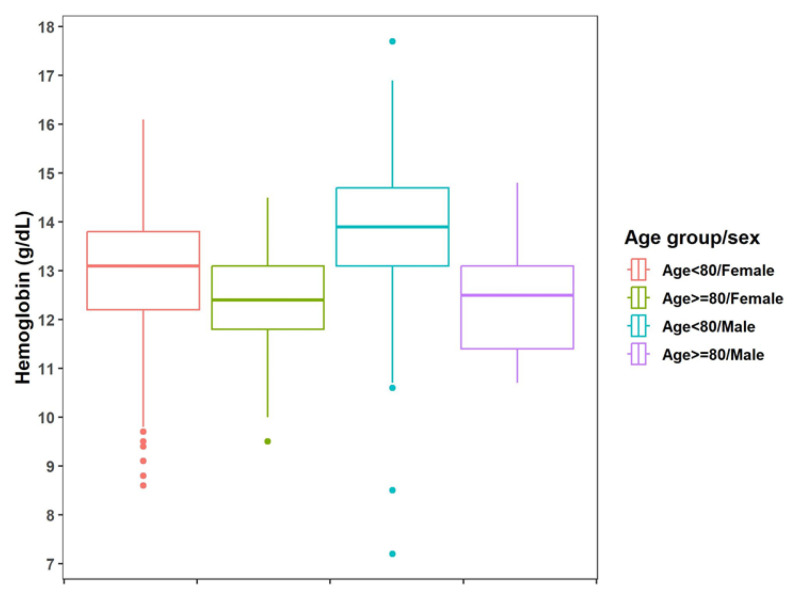
Boxplot of blood hemoglobin dosage, separated by gender and age, among elderly patients with sporotrichosis at INI/Fiocruz from 1999–2020. Patients in group 2 had statistically lower hemoglobin levels (women = *p*-value = 0.003; men = *p*-value = 0.0031).

Concerning the routine laboratory exams, the only parameter that revealed a significant difference between groups 1 and 2 was hemoglobin level. Among the 784 patients with data about this variable, 161 (20.5%) had anemia. When comparing the proportions of patients with laboratory test alterations in the two groups, the tests showed a significantly higher difference in the proportion of patients with anemia (*p* = 0.008) in group 2 (Figure 8). Among the 161 patients with anemia, the majority (*n* = 157, 97.5%) had mild anemia.

### 3.6. Serology for Sporotrichosis

Serology for sporotrichosis was performed for 344 patients, of which 334 (97.1%) were reactive and 10 (2.9%) were non-reactive. Of the latter, all belonged to group 1, were not afflicted with an immunosuppressive condition, and did not use immunosuppressive medication.

### 3.7. Antifungal Treatment, Duration, and Adverse Events

A total of 858 patients were treated with oral antifungals such as itraconazole (administered to 696 patients, constituting 81.1%), terbinafine (administered to 195 patients, constituting 22.7%), and potassium iodide solution (administered to 5 patients, constituting 0.6%).

Itraconazole was used at doses of 100 mg/day for 548 (78.7%) patients, 200 mg/day for 112 patients (16.0%), 300 mg/day for 1 patient (0.1%), and 400 mg/day for 24 patients (3.4%). Eleven (1.6%) patients received itraconazole doses ranging from 100 to 400 mg/day during the evolution of their disease course. Terbinafine was used at doses of 250 mg/day for 183 (93.8%) patients, 500 mg/day for 10 patients (5.1%), and in varied regimens (125, 250, and 500 mg/day) for 2 (1.0%) patients. For 39 (4.5%) patients, there was a need to adjust the dose or switch to another antifungal. Adverse events relating to oral antifungals occurred for 104 (12.1%) patients. The most frequent were nausea/vomiting (*n* = 33, 31.7%), abdominal pain (*n* = 23, 22.1%), and headache (*n* = 12, 11.5%), all of which are considered mild and not requiring suspension.

Amphotericin B was used in eight (0.9%) cases to reinforce the treatment of osteoarticular manifestations (seven) and in a lymphocutaneous case in which the patient did not respond to an oral antifungal (one). Amphotericin B discontinuation occurred in two cases due to azotemia. All patients who used amphotericin B were in group 1.

The median duration of treatment and its range for the different clinical forms was as follows: lymphocutaneous—3.9 (range: 1 to 26.1) months; fixed cutaneous—3.5 (range: 0.7 to 54.7) months; cutaneous disseminated—4.5 (range: 0.7 to 25.2) months; disseminated form—23 (range: 3.3 to 55.7) months; unifocal extracutaneous form—7 (range: 3 to 13.5) months; and contiguous extracutaneous form—23 (range: 3.6 to 19.6) months. There were no differences in the duration of treatment of the clinical forms between groups 1 and 2.

### 3.8. Other Treatments and Procedures

**Figure 9:**
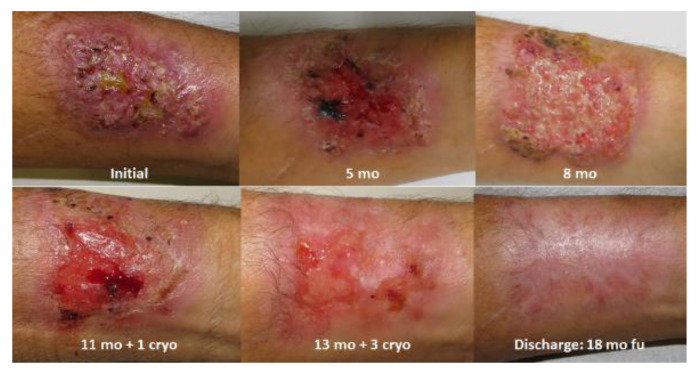
Evolution of fixed sporotrichosis infecting a 64-year-old man. An initially ulcerated, erythematous, infiltrating plaque on the left forearm took 16 months to cure. mo: Months of oral treatment (3 months of itraconazole followed by 10 months of terbinafine). cryo: Cryosurgery with liquid nitrogen spray (total of 4 sessions). fu: Follow-up.

Oral antibiotics were used in 59 (6.5%) cases for the treatment of bacterial secondary infection associated with sporotrichosis lesions. Prednisone was used in 15 (1.6%) cases for the treatment of hypersensitivity syndromes/manifestations. Cryosurgery was performed in 146 (16%) cases (Figure 9), with a median of 3 (IQR: 2–17) sessions. Cryosurgery, as a single treatment for sporotrichosis, was performed in five (0.5%) cases: three due to drug interaction; one due to an adverse reaction to itraconazole before admission to our center (thrombocytopenia); and one due to an infection with chronic hepatitis C. Curettage and electrosurgery were performed in five (0.5%) cases.

### 3.9. Outcomes and Survival Analysis

Clinical cures were achieved in 795 (87.3%) cases, of which 37 (4.1%) were cured spontaneously. Three (0.3%) patients had been undergoing treatment (lymphocutaneous, primary pulmonary, and primary pulmonary + wrist synovitis, with one case *per* patient). Death occurred in seven (0.7%) cases, four of which were caused by issues related to sporotrichosis (three with the disseminated form and one with the pulmonary form), while three patients died due to causes unrelated to mycosis (upper gastrointestinal bleeding, chronic renal failure, or respiratory failure). One hundred and six (11.6%) patients were lost to follow-up.

**Figure 10:**
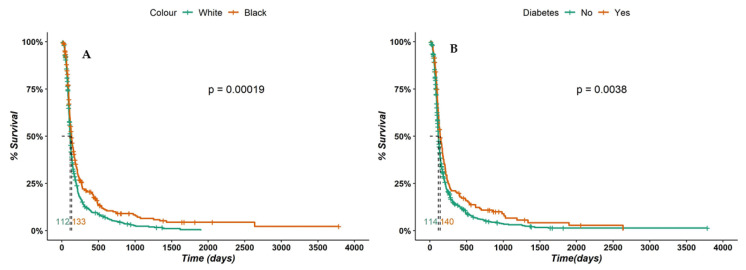
Survival analysis evaluating time of treatment according to (**A**) skin color and (**B**) presence of diabetes.

In the exploratory analysis of survival with respect to the treatment time until achieving a cure that was conducted using the Kaplan–Meier method, the median treatment time until achieving a cure was 119 (IQR 112–126) days. Three variables showed differences between survival curves (*p* < 0.05). White patients were cured in a lower median time (112 days) than black patients (133 days) (*p* < 0.001) (Figure 10A). Patients without diabetes were cured earlier (114 days) than those with diabetes (140 days) (*p* = 0.004) (Figure 10B). Patients without anemia had lower median treatment time (119 days) than those with anemia (159 days) (*p* < 0.001). In the multiple Cox model in which these three variables were controlled with regard to age and clinical form, we observed that diabetes and a black skin color were associated with a longer treatment time until reaching a cure (HR = 0.76 and 0.77, respectively) (Table 3).

### 3.10. Recurrence and Reinfection

Recurrence occurred in 16 (1.8%) cases, occurring after a median of 4.8 (range 1.1 to 19.1) months after antifungal discontinuation. Aside from one patient who was cured after undergoing treatment with cryosurgery, all patients were cured with the reintroduction of oral antifungal medication. Five patients were subsequently treated with cryosurgery along with an oral treatment. Reinfection occurred in 3 (0.3%) cases, with a median of 50.3 (22–73) months after first discharge, and all cases were due to a new exposure to a cat with sporotrichosis.

## 4. Discussion

There are few studies on endemic mycoses in the older population [21]. The increase in life expectancy associated with changes in the epidemiological and clinical aspects of sporotrichosis makes this study important. Figure 1 shows the participation of the older patients in cases of sporotrichosis followed up at the INI over 22 years, which remained proportional in the studied period, even in 2020, the advent of the COVID-19 pandemic, when older people were advised to stay at home.

Older people represent about 12% of the world’s population, and Brazil has the fifth largest older population worldwide [22,23]. In Brazil, those aged 60 years and over are considered older people, while in rich countries, this limit is 65 years. Very old people are considered those 80 years old upwards. This segment is growing due to medical advances and the consequent fall in mortality rates with which they are associated [24]. In this study, although in smaller numbers, people aged up to 95 years were also represented. Women predominated, which is expected of the profile of zoonotic transmission sporotrichosis in Rio de Janeiro, as they more often tend to houses and animals [2,3,4]. Furthermore, female cases were significantly more numerous in the older group. According to Llyod-Sherlock [25], although old age is universally feminine it has a strong gender component.

In general, the frequencies of cutaneous presentations are similar to those described in case series regarding younger people [2,3]. The osteoarticular system was the most affected extracutaneous site (2.1%) due to the contiguity of the cutaneous lesions in the majority of cases. Considering the rarity of these presentations, we believe that this value is expressive [26]. Among older people, the skin barrier is weakened due to the reduction in its layers and components [27,28]. In this context, the progression of fungal infection may occur beyond the skin, and this is also supported by a larger and more virulent inoculum, which is typical of zoonotic transmission [29]. The most common manifestation observed was osteomyelitis, but our attention was also drawn to nodules and/or masses on the backs of the hands, which are manifestations corresponding to synovitis/tenosynovitis. These unusual lesions may be in part due to degenerative changes in the tendons, whose elastic capacity, strength, and resistance decrease with age [30]. Primary pulmonary sporotrichosis, rarely described in the hyperendemic state of Rio de Janeiro, is related to previous pulmonary alterations, which present in three of our patients [31,32]. The involvement of the ocular and nasal mucosa is compatible with this form of transmission [2,3].

In this study, a small proportion of patients had immunosuppressive conditions or used immunosuppressive drugs that, combined with immunosenescence, could lead to severe forms such as meningeal sporotrichosis, as described in *S. brasiliensis* infection [33,34,35]. However, all patients had cutaneous forms of the infection, except those who abused alcohol. We emphasize the importance of controlling the underlying disease and investigating the dissemination of this mycosis to detect oligo/asymptomatic lesions on immunosuppressed patients [36].

In our study, the manifestations of hypersensitivity, another characteristic of *S. brasiliensis* infection, were observed at a statistically significant lower frequency among patients aged 80 years or older [29,37]. However, when we compared our patients with a cohort of patients with hypersensitivity reactions from the hyperendemic region in Rio de Janeiro (8.1%), we observed that older patients had a lower frequency (2.7%) [37]. These reactions are immune-mediated, involving the participation of T lymphocytes and cytokines, whose functions are altered due to immunosenescence [13,14].

Serology for sporotrichosis is based on antibody detection (mainly IgG) tests and provides a presumptive diagnosis of this mycotic disease [38]. In this study, this test showed an excellent sensitivity (97.1%) with respect to the study population, although in cases of immunosenescence, there may be a reduction in the number of B cells with a decrease in antibody production, as seen, for example, in the decrease in the immune response to many vaccines [39,40].

The older population investigated presents comorbidities and uses medications, both of which can contribute to the likelihood of suffering from more severe infections and experiencing adverse drug reactions. However, both itraconazole and terbinafine were effective and well tolerated, with few adverse reactions [41,42,43]. As most of our population consists of low-income individuals, we attribute this good response to the availability of two antifungals free of charge at the INI pharmacy. Terbinafine is always indicated when patients use many medications (known as polypharmacy, i.e., the use of five or more medications) or for those presenting drug interactions with itraconazole [41,43]. In addition, most patients responded to standard low doses of the antifungal used (itraconazole 100 mg/day and terbinafine 250 mg/day). When we compare the treatment times of the main clinical forms in our study (lymphocutaneous and fixed cutaneous) with studies with the same epidemiological characteristics but involving a younger population, it took our patients approximately one month longer until they were cured [2,42]. We believe that the best option for older patients with sporotrichosis, who do not have serious complications due to the disease, is to “start low and go slow”, that is, to be administered low doses of antifungals with longer treatment times [44]. In this context, we highlight the role of adjuvant or isolate cryosurgery as an excellent tool in the treatment of these patients [20]. The treatment of the extracutaneous forms required more time, as expected [18]. Spontaneous remission was also observed in this population, as previously described for sporotrichosis [2,3].

Irrespective of the drug regimen, 87.3% of the cases were cured, and four patients died of sporotrichosis, reinforcing the opportunistic form that this mycosis can present [9,10,35]. Black patients and patients with diabetes required the most time until they were cured. In Brazil, there is a historical socioeconomical discrepancy that keeps the black population in a situation of greater economic vulnerability for which there is greater difficulty in accessing referral health services [45,46]. Diabetes mellitus, the second most common comorbidity in this study after systemic arterial hypertension, is associated with macro- and microangiopathies, which delay the healing process, as well as altered mechanisms of response to infections [47]. More clinical and epidemiological studies should be carried out to better understand the influence of these variables in relation to the duration of treatment for sporotrichosis.

Although the observed relapses were similar in number to those already described, we should be careful when discontinuing the administration of antifungal agents, as older patients may not show obvious inflammatory signs on their skin, which can be misconstrued as the healing of lesions. Considering that most patients continue to live with cats in hyperendemic areas, reinfections were low in number, which may be due to the fact that some immunity to sporotrichosis must be conferred through corresponding interactions [2].

## 5. Conclusions

In this study, older patients presented the usual clinical forms of sporotrichosis. We have drawn attention to the frequency of osteoarticular involvement secondary to skin lesions. Cures were achieved in most cases through the use of itraconazole or terbinafine in low standard doses coupled with cryosurgery. Black patients and those with diabetes had longer treatment times. Therefore, these two subgroups of patients should be monitored more closely to reduce possible difficulties that may arise during treatment. It would be interesting for more studies to be conducted on older patients with sporotrichosis from different epidemiological and geographic areas in order to better understand the disease in such hosts.

## Figures and Tables

**Table 1:** Clinical and demographic data of older adult patients with sporotrichosis from a hyperendemic area of zoonotic transmission in Rio de Janeiro, Brazil.

Variables	Status	*n* = 911 (100%)
Sex	Female	666 (73.1)
	Male	245 (26.9)
Skin color	White	530 (62.1)
	Black ^a^	323 (37.9)
Clinical presentation	Lymphocutaneous	562 (62)
	Fixed cutaneous	233 (25.7)
	Cutaneous disseminated	81 (8.9)
	Extracutaneous/Disseminated ^b^	30 (3.3)
Organs affected ^c^	Skin	900 (99.3)
	Osteoarticular	19 (2.1)
	Ocular	6 (0.7)
	Upper airways	3 (0.3)
	Lungs	3 (0.3)
Hypersensitivity reactions		25 (2.8)
Comorbidities (number)	1	376 (43.1)
	2	191 (21.9)
	3	52 (5.9)
	≥4	17 (1.9)
Treatment	Itraconazole	696 (81.1)
	Terbinafine	195 (22.7)
	Iodide solution	5 (0.5)
	Amphotericin	8 (0.9)
Outcome	Cure	795 (87.3)
	Death due to sporotrichosis	4 (0.4)
	Death due to other causes	3 (0.3)
	Still under treatment	3 (0.3)
	Lost to follow-up	106 (11.6)

^a^ black = mixed-black and black; ^b^ extracutaneous/disseminated = unifocal extracutaneous (single site of involvement or two contiguous sites) and multifocal/disseminated (two or more non-contiguous sites) forms; ^c^ 22 patients had more than one organ affected. Missing: skin color = 58; clinical presentation, organs affected, and hypersensitivity reactions = 5; comorbidities = 38.

**Table 2:** Extracutaneous/multifocal disseminated clinical forms of the patients with sporotrichosis enrolled in this study.

Variables	Status	
Unifocal extracutaneous sporotrichosis	ocular mucosa	4
	nasal mucosa	2
	pulmonary	2
	arthritis	1
Contiguity unifocal extracutaneous sporotrichosis	lymphocutaneous + osteomyelitis	2
	fixed cutaneous + osteomyelitis	2
	lymphocutaneous + osteomyelitis + synovitis	1
	lymphocutaneous + synovitis	3
Multifocal/disseminated sporotrichosis	disseminated skin lesions + osteomyelitis	6
	disseminated skin lesions + osteomyelitis + tenosynovitis	2
	lymphocutaneous + ocular mucosa	2
	lymphocutaneous + nasal mucosa	1
	primary pulmonary sporotrichosis + wrist synovitis	1
	arthritis (in the knee and wrist)	1

Total = 30 patients.

**Table 3:** Multiple Cox regression model of possible predictors for the treatment times of older patients with sporotrichosis, INI/FIOCRUZ (1999–2020).

Variables/Categories		HR	(95% CI) *	*p*-Value	aHR	(95% CI) *	*p*-Value
Black skin color	Yes	0.74	(0.64–0.86)	*p* = 0.001	0.74	(0.63–0.87)	*p* < 0.001
Diabetes mellitus	Yes	0.77	(0.64–0.92)	*p* = 0.004	0.76	(0.62–0.93)	*p* = 0.007
Anemia	Yes	0.80	(0.66–0.97)	*p* = 0.023	0.84	(0.69–1.03)	*p* = 0.086
Age ≥ 80 years old	Yes	0.87	(0.68–1.12)	*p* = 0.286	0.83	(0.62–1.10)	*p* = 0.187

Model adjusted for clinical form (lymphocutaneous and fixed cutaneous, disseminated cutaneous and extracutaneous/disseminated). HR = hazard ratio; aHR = adjusted hazard ratio; * CI = confidence interval. Values in bold are those with a statistically significant difference at the 5% level.

## Data Availability

All relevant data are presented in the article.

## Supplementary Materials

The following supporting information can be downloaded at: https://www.mdpi.com/article/10.3390/jof9080804/s1, Table S1. Comorbidities of older patients with sporotrichosis treated at INI/FIOCRUZ (1999–2020).
