# Supplementary material for: Sporotrichosis in Older Adults: A Cohort Study of 911 Patients from a Hyperendemic Area of Zoonotic Transmission in Rio de Janeiro, Brazil
Source: J Fungi (Basel). 2023 Jul 30;9(8):804. doi: 10.3390/jof9080804 (PMC10455193; doi:10.3390/jof9080804)
Supplement: Supplementary file 1 [file jof-09-00804-s001.zip › jof-2498164-Supplementary Materials.pdf]

Table S1. Comorbidities of older patients with sporotrichosis treated at INI/FIOCRUZ (1999-2020)

Comorbidities	Frequency	Percentage
**Systemic arterial hypertension**	478	75.2
**Diabetes mellitus**	163	25.6
**Dyslipidemia/metabolic changes**	48	7.5
**Cardiovascular diseases [1]**	46	7.2
**Arthrosis/osteoporosis**	34	5.3
**Neuropsychiatric diseases [2]**	32	5.0
**Gastrointestinal diseases [3]**	23	3.6
**Hypothyroidism**	23	3.6
**Nephropathy/uropathies [4]**	19	3.0
**Chronic obstructive pulmonary disease/asthma**	18	2.8
**Glaucoma**	17	2.7
**Chronic venous/lymphatic insufficiency**	12	1.9
**Neoplasms [5]**	10	1.6
**Infectious diseases [6]**	7	1.1
**Lupus erythematosus/rheumatoid arthritis**	6	0.9
**Alcoholism**	5	0.8
**Haematological diseases [7]**	5	0.8
**Skin diseases [8]**	3	0.5

[1] Coronary disease, heart failure, arrhythmia, heart disease. [2] Anxiety, convulsions, depression, psychiatric disease, Parkinson's disease. [3] Gastroesophageal reflux disease, cirrhosis, gastritis, peptic ulcer. [4] Chronic renal failure, nephrolithiasis, benign prostatic hyperplasia. [5] Cervical cancer, breast cancer, prostate cancer. [6] Chagas disease, human immunodeficiency virus infection, human T-cell lymphotropic virus infection, hepatitis C virus infection. [7] Myeloid leukemia, idiopathic thrombocytopenic purpura. [8] Neurofibromatosis, psoriasis, vitiligo. Missing = 38.
